# Vitamin D deficiency and length of pediatric intensive care unit stay: a prospective observational study

**DOI:** 10.1186/s13613-015-0102-8

**Published:** 2016-01-08

**Authors:** Jhuma Sankar, Wonashi Lotha, Javed Ismail, C. Anubhuti, Rameshwar S. Meena, M. Jeeva Sankar

**Affiliations:** Department of Pediatrics, All India Institute of Medical Sciences, New Delhi, India; Department of Pediatrics, PGIMER, Dr RML Hospital, New Delhi, India; Department of Biochemistry, PGIMER, Dr RML Hospital, New Delhi, India

**Keywords:** Vitamin D deficiency, 25 (OH) D deficiency, Prevalence, Critically ill, Vitamin D, 25 (OH) D, Tropical country, Duration of PICU stay

## Abstract

**Background:**

Due to the limited data available in the pediatric population and lack of interventional studies to show that administration of vitamin D indeed improves clinical outcomes, opinion is still divided as to whether it is just an innocent bystander or a marker of severe disease. Our objective was therefore to estimate the prevalence of vitamin D deficiency in children admitted to intensive care unit (ICU) and to examine its association with duration of ICU stay and other key clinical outcomes.

**Methods:**

We prospectively enrolled children aged 1 month–17 years admitted to the ICU over a period of 8 months (*n* = 101). The primary objectives were to estimate the prevalence of vitamin D deficiency (serum 25 (OH) <20 ng/mL) at ‘admission’ and to examine its association with length of ICU stay.

**Results:**

The prevalence of vitamin D deficiency was 74 % (95 % CI: 65–88). The median (IQR) duration of ICU stay was significantly longer in ‘vitamin D deficient’ children (7 days; 2–12) than in those with ‘no vitamin D deficiency’ (3 days; 2–5; *p* = 0.006). On multivariable analysis, the association between length of ICU stay and vitamin D deficiency remained significant, even after adjusting for key baseline variables, diagnosis, illness severity (PIM-2), PELOD, and need for fluid boluses, ventilation, inotropes and mortality [adjusted mean difference (95 % CI): 3.5 days (0.50–6.53); *p* = 0.024].

**Conclusions:**

We observed a high prevalence of vitamin D deficiency in critically ill children in our study population. Vitamin D deficient children had a longer duration of ICU stay as compared to others.

**Electronic supplementary material:**

The online version of this article (doi:10.1186/s13613-015-0102-8) contains supplementary material, which is available to authorized users.

## Background

Vitamin D deficiency is common and has been estimated to affect about one billion people worldwide [1]. While the primary role of this pleiotropic hormone is regulation of calcium metabolism, it also plays a key role in several pathways of the innate immune response system, controlling cell growth, differentiation and apoptosis [1], [2]. About 90 % of vitamin D needs are met with adequate sun exposure while dietary sources contribute only to the remaining 10 % of daily requirement [1].

It is well known that any critical illness or injury disturbs the internal milieu of the body and may affect the reserves of vital nutrients and minerals of the body. Nutrition is often suboptimal in these patients and this may further aggravate any deficiencies. Vitamin D deficiency has been reported to be widely prevalent in critically ill children from ICUs worldwide [3–6]. According to these studies, the prevalence of vitamin D deficiency has ranged anywhere from 30 to 70 % [3–6]. Although one would expect that in the tropics with greater sun exposure, vitamin D deficiency would be less as compared to that from the temperate regions of the world, the prevalence reported from the tropical countries is similar [6–10]. In one of the largest pediatric studies published till date, the authors observed lower vitamin D levels in those over weight as compared to underweight children [4]. Whether this holds true in patients admitted to resource poor settings where the burden of undernourished children is much higher is yet to be evaluated. Such data would be useful in designing clinical trials targeting this important subgroup of critically ill children.

Whether vitamin D deficiency affects illness severity and clinical outcomes has been the subject of much debate in the pediatric as well as the adult population. While in few of these studies, it has been associated with greater illness severity at presentation, increased need for inotropes, mechanical ventilation, duration of stay and even mortality [3–6, 11, 12] in few others no such association was found [13, 14]. Due to the limited data available in the pediatric population and lack of interventional studies to show that administration of vitamin D indeed improves clinical outcomes, opinion is still divided as to whether it is just an innocent bystander or a marker of severe disease. Further evidence from the pediatric population and interventional studies is therefore needed to address this dilemma.

With this background, we hypothesized that the prevalence of vitamin D deficiency would be high in critically ill children admitted to our ICU and that this deficiency would be associated with clinically important outcomes in these children.

## Methods

### Design and setting

We conducted this prospective observational study over a period of 8 months (July–Dec 2013) in children admitted to the pediatric intensive care unit (PICU) of our tertiary care centre.

### Participants

All critically ill children aged ≤17 years (1 month–≤17 years) admitted to PICU were enrolled till the estimated sample size was met. We excluded children who were already on vitamin D supplementation, had received large doses for rickets or documented vitamin D deficiency in the past 1 year or steroids for at least 10 days before admission, or had recent kidney stones or chronic kidney disease. Eligible children were enrolled in the study after obtaining informed written consent from parents. The study was approved by the Institutional Ethics Committee.

### Objectives and outcome measures

Our primary objectives were to estimate (1) the prevalence of vitamin D deficiency, defined as serum 25 (OH) D ≤ 20 ng/mL [15] and (2) the association between vitamin D deficiency and length of ICU stay. Our secondary objectives were to (1) evaluate the prevalence of vitamin D deficiency in moderately undernourished and severely undernourished children [16] and (2) examine the association between vitamin D deficiency and other important variables such as Pediatric Logistic Organ Dysfunction (PELOD) score at 24 h, need for fluid boluses during first 6 h, need for mechanical ventilation and inotropes, and mortality. The definitions used for the purpose of the study are provided in panel 1 (Additional file 1: Table S1).

## Methods

The children were managed as per preexisting protocols for management for various conditions. We followed a uniform protocol of nutritional support for all children admitted in PICU [17] irrespective of their underlying nutritional status in the acute phase of their illness. Calories and proteins for growth were increased as per their recommended dietary allowance (RDA) once we could achieve full feeds in these children. And once we achieved full feeds, within a day or two they were shifted to the step down unit where their growth was monitored till their discharge. We did not use routine supplementation of vitamin D in any of the children. Data were recorded on a pre-specified data collection form which included demographic details, illness severity score (Pediatric index of mortality-2 or PIM-2) at admission, duration of sun exposure (determined by questioning the parents as to the number of hours the child stayed outdoors on an average per day) and clinical details on a daily basis till death or discharge from the hospital. Relevant laboratory tests were performed on all patients at admission. Arterial lactate, ionized calcium, parathyroid hormone were measured at inclusion. Samples for estimation of serum 25 (OH) D levels were drawn at admission (within the first hour) alongside other blood tests. Samples were cold centrifuged at 4 °C and the plasma aliquoted and stored at −20 °C till sufficient samples were collected to run the test. Serum 25-hydroxyvitamin D was measured with automated chemiluminescent immunoassay technology (VITROS eci, Johnson and Johnson Ortho Clinical Diagnostics). The analytical sensitivity of this test is 4 ng/mL for 25 (OH) D with a reportable range of 4–512 ng/mL.

### Sample size estimation

We calculated the sample size for the first primary objective—prevalence of vitamin D deficiency. Assuming the prevalence of vitamin D deficiency to be 50 %, a confidence level of 95 %, absolute precision of 10 %, and design effect of 1, the sample size required was 97.

### Statistical analysis

Data were entered into Microsoft Excel 2007 and analyzed using Stata 11.2 (Stata Corp, College Station, TX). Results are presented as mean (SD) or median (interquartile range) as appropriate for continuous variables and as absolute numbers (%) for categorical variables. For determining association between vitamin D deficiency and demographic and key clinical outcomes, we performed univariable analysis using Student’s *t* test/Wilcoxon rank-sum test and chi-square test for continuous and categorical variables, respectively. As our primary objective was to study the association between vitamin D deficiency and length of stay, we performed multivariable regression analysis with length of stay as the dependant variable after adjusting for important baseline variables such as age, gender, PIM-2, PELOD, weight for age, diagnosis and, outcome variables like mechanical ventilation, inotropes, need for fluid boluses in first 6 h and mortality. The selection of baseline variables was before the start of the study. We used clinically important variables irrespective of *p* values for the multivariable analysis. The results of the multivariable analysis are reported as mean difference with 95 % confidence intervals (CI).

## Results

**Fig. 1:**
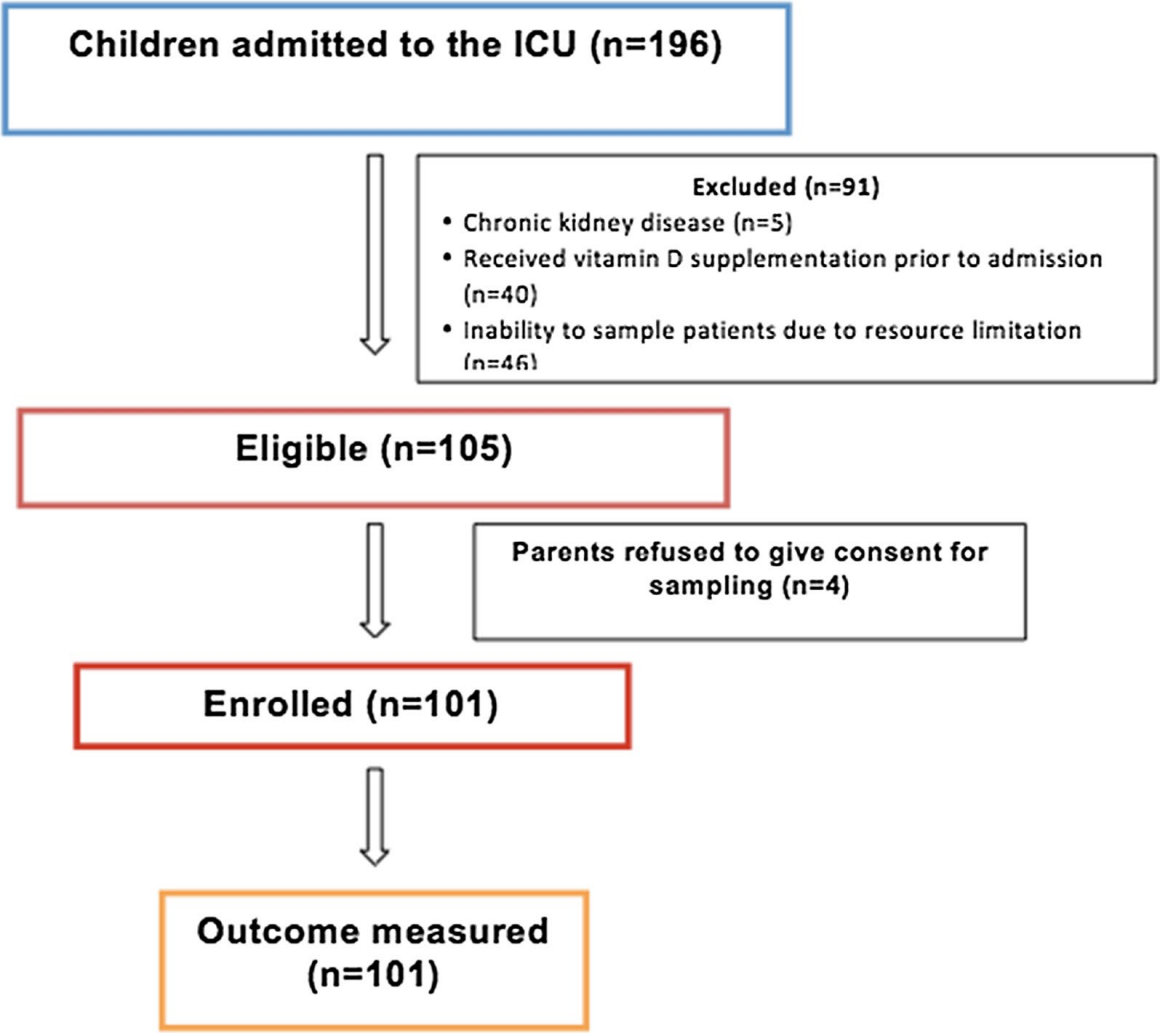
Study flow chart

A total of 196 children were admitted to the ICU during the study period. Of these 95 were excluded as per pre-specified exclusion criteria (Fig. 1) and inability to sample patients for 2 months (September and October) due to logistic reasons. Baseline demographic and clinical data are described in Table 1. The median age was 3 years (IQR 0.1–9) and there was a slight preponderance of boys (52 %). The median (IQR) PIM-2 probability of death (%) at admission was 12 (8–26) and PELOD score at 24 h was 21 (20–22). About 40 % were admitted during the winter season (Nov–Dec). The most common admitting diagnosis was pneumonia (19 %) and septic shock (19 %). Fifteen children had features of hypocalcemia at admission.

**Table 1:** Baseline demographic and clinical characteristics of children enrolled in the study

Variable	*n* = 101
Age (median, IQR)	3 (1 months, 9 years)
<1 year	25 (25)
1–5 years	33 (33)
6–10 years	26 (26)
11–17 years	17 (17)
Male (*n* %)	52 (52)
PIM-2 score (median, IQR)	12 (8–26)
PELOD score at admission (median, IQR)	21 (20–22)
Weight (Kg), median (IQR)	12 (5–19)
Duration of sun exposure in hours/day (only exposed parts) (median, IQR)	2 (0.5–3.5)
Admission season (*n* %)	
Nov–Dec	38 (38)
Rest of the year	63 (63)
Nutritional status (*n* %)	
Normal	32 (31.7)
Moderately undernourished (−2 to −3 SD)	39 (38.6)
Severely undernourished (<−3 SD)	30 (29.7)
Admitting diagnosis, *n* (%)	
Severe sepsis/septic shock	19 (19)
Pneumonia	19 (19)
Meningitis	16 (16)
Seizure disorder	12 (12)
Cardiac illness	10 (10)
Tuberculosis	3 (3)
Malaria	3 (3)
Hepatic failure	2 (2)
Raised ICP	1 (1)
Any other	16 (16)
Underlying illness (*n* %)	
Congenital heart disease	9 (9)
Nephrotic syndrome	3 (3)
Genetic/neurometabolic disorders	3 (3)
Tubercular meningitis	1 (1)
Others including autoimmune/immunodeficiency disorders	4 (4)
Neurological illness	15 (15)
Symptomatic hypocalcemia at admission (*n* %)	15 (15)
Laboratory investigations [mean (SD) or median (IQR)]	
Total calcium (mg/dL)	8 (1)
Phosphate (mg/dL)	3.3 (0.5)
Ionized calcium (mmol/L)	0.65 (0.25)
Alkaline phosphatase (IU/L)	159 (123–343)
SGOT (U/L)	57 (34–191)
SGPT (U/L)	39 (22–114)
Albumin (g/dL)	2.9 (0.4)
Creatinine (mg/dL)	0.6 (0.4–0.7)
Hemoglobin (g/dL)	9.7 (2)

*IQR* interquartile range*, PELOD* pediatric logistic organ dysfunction*, PIM* pediatric index of mortality*, CI* confidence interval*, ICP* intracranial pressure*, SGOT* serum glutamic oxaloacetic transaminase*, SGPT* serum glutamic-pyruvic transaminase

The prevalence of vitamin D deficiency was 74 % (95 % CI: 65–88) (Table 2) with a median serum vitamin D level of 5.8 ng/mL (IQR: 4–8) in those deficient. Sixty one % (*n* = 62) had severe deficiency (levels <15 ng/mL) [18]. The prevalence of vitamin D deficiency was 80 % (95 % CI: 66–93) in children with moderate under-nutrition while it was 70 % (95 % CI: 53–87) in those with severe under-nutrition (Table 2). The median (IQR) serum 25 (OH) D values for moderately undernourished, severely undernourished, and in those without under-nutrition were 8.35 ng/mL (5.6, 18.7), 11.2 ng/mL (4.6, 28), and 14 ng/mL (5.5, 22), respectively. There was no significant association between either the prevalence of vitamin D deficiency (*p* = 0.63) or vitamin D levels (*p* = 0.49) and the nutritional status.

**Table 2:** Prevalence of vitamin D deficiency at admission

	All children (A)	Normal nutritional status (B)	Moderate under-nutrition^a^ (C)	Severe under-nutrition^b^ (D)	P value between (B), (C) and (D)
Prevalence * n*/*N*; %, (95 % CI)	75/101	24/32 76 (58–94)	31/39 80 (66–93)	21/30 70 (53–87)	0.63
Vitamin D levels at admission in deficient children (median, IQR)	5.8 (4–8)	14 (5.5–22)	8.35 (5.6–18.7)	11.2 (4.6–27.7)	0.49

^a^Weight for age <−2 SD

^b^Weight for age <−3 SD

On evaluating the association between vitamin D deficiency and important demographic and clinical variables, children with vitamin D deficiency were found to be older (median age, 4 vs. 1 years), and were more likely to receive mechanical ventilation (57 vs. 39 %) and inotropes (53 vs. 31 %) (Table 3). None of these associations were, however, statistically significant.

**Table 3:** Comparison of demographic and clinical variables between vitamin D deficient and ‘not deficient’ groups

Outcome variables	Vitamin D deficiency *N* = 75	‘No deficiency’ *N* = 26	*P* value
Age (yrs)	4 (0.5–9)	1 (0.4–8)	0.12
Female gender	39 (53)	10 (37)	0.16
Weight for age			
Moderate under-nutrition	31 (80)	8 (31)	0.33^*^
Severe under-nutrition	21 (70)	9 (34)	0.66^*^
PIM2-probability of death (%) (median, IQR)	12.5 (8.6–23.5)	11.5 (6.8–30)	0.45
PELOD score (median, IQR)	21 (11–22)	21 (10–21)	0.09
Diagnosis (infections)	47 (64)	16 (59)	0.69
Hypocalcemia			
Total	27 (36)	6 (37.5)	0.9
Ionized	58 (77)	15 (94)	0.14
Serum calcium (median, IQR)			
Total (mg/dL)	8.5 (7.4–8.9)	8.7 (7.1–9.2)	0.30
Ionized (mmol/L)	0.65 (0.4–0.8)	0.70 (0.63–0.9)	0.21
Parathyroid levels (pg/mL)	16 (1.4)	16.6 (5)	0.36
Need for fluid boluses in first 6 h	44 (59)	12 (38)	0.14
Need for mechanical ventilation (*n* %)	43 (57)	10 (39)	0.10
Duration of ventilation (days)			
Median (IQR)	6.5 (3.5–14)	7 (2–13)	0.55
Need for inotropes (*n* %)	40 (53)	8 (31)	0.06
Inotrope score	1320 (960–7040)	2440 (1440–3120)	0.23
Duration of inotrope therapy, days (median, IQR)	2 (2–4)	1.5 (1–2)	0.15
Duration of PICU stay	7 (2–12)	3 (2–5)	0.006
Mortality (*n* %)	23 (31)	8 (31)	1.0

Data presented as number (proportion), mean (SD), or median (IQR)

*PELOD* pediatric logistic organ dysfunction, *PIM* pediatric index of mortality, *CI* confidence interval, *IQR* interquartile range, *PICU* pediatric intensive care unit

^*^ Compared to no under-nutrition

**Fig. 2:**
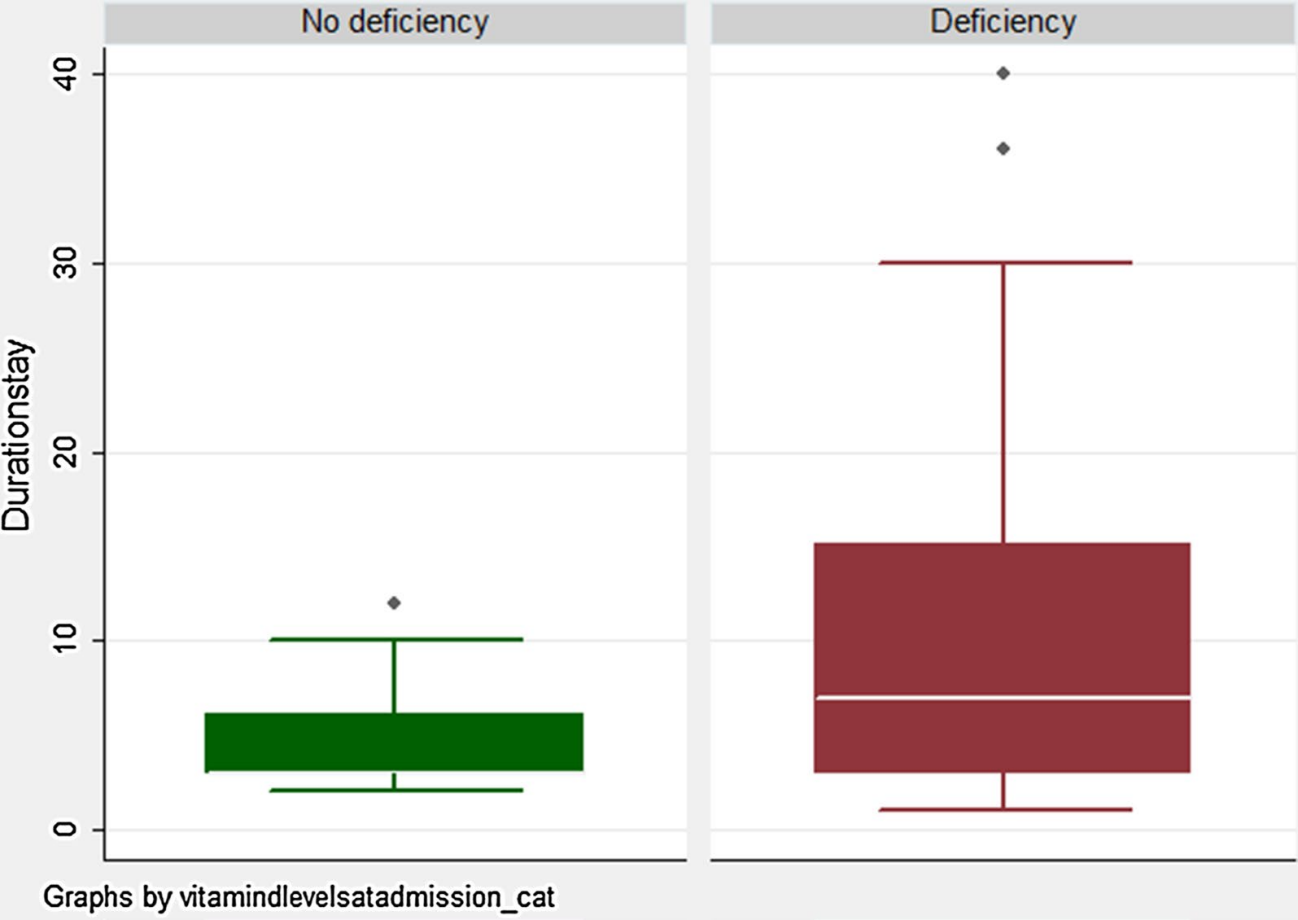
Association between vitamin D deficiency and length of ICU stay

The median (IQR) duration of ICU stay was significantly longer in vitamin D deficient children (7 days; 2–12) than in those with no vitamin D deficiency (3 days; 2–5; *p* = 0.006) (Fig. 2). On multivariable analysis, the association between length of ICU stay and vitamin D deficiency remained significant, even after adjusting for key baseline variables, diagnosis, illness severity (PIM-2), PELOD, and need for fluid boluses, ventilation, inotropes, and mortality [adjusted mean difference (95 % CI): 3.5 days (0.50–6.53); *p* = 0.024] (Table 4).

**Table 4:** Multivariable regression for association between length of stay and vitamin D deficiency after adjusting for key baseline and clinical variables

Variables	Mean difference (95 % CI)	P value
Vitamin D deficiency	3.50 (0.50–6.53)	*0.024*
Age	0.007 (−0.01–0.03)	0.51
Gender (male)	0.99 (−1.60–3.59)	0.44
PIM-2	0.006 (−0.05–0.07)	0.84
PELOD	0.05 (−0.14–0.24)	0.61
Diagnosis (infections vs. others)	0.49 (−2.25–3.25)	0.72
Mortality	0.06 (−4.07–3.94)	0.97
Need for mechanical ventilation	1.26 (−1.42–3.95)	0.35
Need for inotropes	3.85 (−2.10–9.89)	0.20
Need for fluid boluses in first 6 h	0.39 (−5.79–5.00)	0.88

*PELOD* pediatric logistic organ dysfunction score, *PIM* pediatric index of mortality, *CI* confidence interval

## Discussion

Our data suggests a high prevalence (74 %) of vitamin D deficiency in our study population. In two recently published studies from India, the prevalence in critically ill children in general was found to be 40 % [6] and in children with sepsis it was around 50 % [10]. Despite being from a tropical country, the incidence of vitamin D deficiency in our study is as high as has been reported from temperate countries such as in the study by Madden et al. [3]. While Madden et al. attributed the high incidence in the critically ill population in their study to factors such as transcapillary leak, fluid administration, and organ dysfunction, the high incidence observed in ours could be attributed to the high incidence of underlying deficiency in the general population as such in addition to the factors described above in critically ill children. Various factors like duration and timing of sun exposure, amount of skin exposed, skin pigmentation, dietary and genetic factors [19, 20], have been implicated as possible reasons for the high incidence of vitamin D deficiency observed in the tropics. However, as most cases in the general population are asymptomatic therefore, the deficiency may not be clinically relevant. The same cannot be said for the critically ill population where there are multiple other factors involved and the physician is only trying to optimize various therapeutic options to decide what works best for the patient. Therefore, deficiency of any essential nutrient or element deserves careful consideration.

Though high prevalence of vitamin D deficiency in critically ill adults and children has been documented, the impact of such deficiency is not yet clear. Few studies have documented significant association of deficiency with poor outcomes such as longer duration of ICU stay [4, 21], increased inotropic requirement [5, 11] and higher admission illness severity scores [3, 4]. We chose length of ICU stay as a clinically important outcome for the primary objective as it was one of the key variables found to be significantly associated with vitamin D deficiency in the largest prospective study of 326 children published till date [4]. On multivariable regression analysis, a value of less than 50 nmol/L (20 ng/mL) was independently associated with a PICU stay of an additional 1.92 days (95 % CI: 0.2–3.7; *p* = 0.03) in this study [4]. In our study too, we observed that the length of ICU stay was longer in children with ‘vitamin D deficiency’ with a mean difference in PICU stay of 3.5 days (95 % CI: 0.50–6.53; *p* = 0.024) as compared to those ‘not deficient’. The association remained significant even after adjusting for key baseline and clinical variables. In comparison to the study by Mc Nally et al., the overall duration of stay as well as the difference in length of stay was greater in our study between ‘deficient’ and ‘not deficient’ children despite having similar incidence rates as in their study. Differences in patient population such as medical or surgical patients, age, genetic heterogeneity, underlying nutrition status and admitting diagnosis could be the possible reasons for the longer duration of stay as compared to their study. For example, in the study by Mc Nally et al., the study population was mostly surgical (70 % of patients) (cardiac or otherwise) whereas our population was only medical. Medical conditions have a long drawn course of illness unlike surgical patients in who the recovery is quicker and patients are shifted out of the ICU once their post-op period is uneventful and they are extubated [4]. The mean duration of mechanical ventilation (intubation) was 3.5 days in their study compared to 9 days in our study in the deficient children. Even in the other group it was 2.6 days in their study compared to 8 days in our study. Deficient children were older as compared to those ‘not deficient’. This could have been due to lack of exposure to adequate sunlight during school hours or very little exposure owing to mostly indoor activities in this age group apart from dietary factors. Moreover, there were large numbers of undernourished children in our study population which may have contributed to longer diseases course and slower recovery in these children and therefore prolonged the stay in both groups and in the deficient group much more. The undernourished children with vitamin D deficiency could have had other micronutrient/essential nutrient deficiency which were not overtly manifesting but could have contributed to the illness severity and therefore the slow recovery. The admitting diagnoses were mostly infections in our study compared to cardiac and non-cardiac surgical cases in their study. In a previous study from our Institute, we observed that the median duration of stay in children with infections with or without shock is 5–7 days [22]. Thus, although the duration is longer compared to the study by Mc Nally et al., the differences in the patient population, clinical course and outcomes in these two settings might be contributory.

Vitamin D deficiency is presumed to increase morbidity and mortality by its pleiotropic effects on various organ system functions and its effects on innate and adaptive immunity [2, 12, 23]. Although a cause and effect relationship has not been clearly established due to the conflicting evidence available, it is presumed that deficiency of this hormone may contribute to triggering or aggravating the multi organ dysfunction that occurs in the critically ill and is responsible for the increased morbidity and mortality in this population [3, 4, 23]. Although the differences were not statistically significant on univariable analysis, we observed that children who were vitamin D deficient at admission were more likely to require mechanical ventilation, inotropes, fluid boluses, have higher organ dysfunction scores and have prolonged duration of mechanical ventilation. All these factors in combination might have contributed to the slow recovery in this group of children thereby prolonging the length of stay in these patients.

There is limited data available in pediatric literature on the effect of vitamin D deficiency on clinically important outcomes and there is no evidence to support that outcomes may improve with supplementation. Supplementation of vitamin D in cases of deficiency is therefore, only subjective and should be based on features of deficiency (such as due to hypocalcemic seizures, bowed legs, rickets on X-ray) [24] rather than only the serum levels. A large randomized control trial (VITdAL-ICU) [25] in critically ill adults involving vitamin D supplementation showed no significant difference in length of ICU stay or other outcomes after supplementation with vitamin D3 against placebo group. Presently, there is no recommendation for routine supplementation in adults too in view of inconclusive evidence. Therefore, there is a need to generate further evidence on the role of supplementation in children.

Twenty-five (OH) D being the stable storage form, estimation of its levels in serum reflects the total body stores and thus is widely accepted parameter for assessment of vitamin D status. The most commonly and widely used definition of vitamin D deficiency used to estimate prevalence in several populations around the world has been a cut off value of 25(OH) D of <20 ng/mL or <50 nmol/L and is also recommended by the American Academy of Pediatrics (AAP) committee on nutrition [26] and US Endocrine Society [15] for starting therapy in symptomatic cases. Therefore, we used this definition as has been used in similar previous studies in children [3, 4]. A level of <37.5 nmol/L or <15 ng/mL indicates severe deficiency and may manifest clinically as rickets and/or histologically as osteomalacia [18, 27, 28]. The number of children with severe deficiency in our study was 62 (61 %). Severe deficiency like mild-moderate deficiency has been shown to be associated with increased disease severity, duration of stay as well as mortality in few while no association has been found in few other studies [29, 30, 31]. Most interventional studies have used 20 ng/mL as the cut off for supplementation [24]. However, it appears that the group of patients to benefit the most would be the ones with severe deficiency and therefore interventional studies should be planned targeting this group rather than those with any deficiency.

We anticipated a higher prevalence of vitamin D deficiency in malnourished children due to poor diet, altered metabolism or reduced environmental ultraviolet exposure. Although the median 25(OH) D levels were lower in the malnourished group, the prevalence of vitamin D deficiency was observed to be high in both well-nourished and malnourished groups. Similar observation has been documented in the study by Mc Nally et al. [4] where the authors observed that, children with higher weight for age were more deficient than others. The reasons for this observed phenomenon could be the same as described for deficiency in healthy school children such as lack of exposure to sunlight due to staying indoors, increased skin pigmentation and unknown genetic factors [7, 9]. The median vitamin D levels were higher in the severely malnourished group as one child had a level of 54 mmol/L which increased the overall median in this population. Vitamin D supplementation at some point in time (1 year before enrollment into the study) was probably responsible for high levels in this child although severely undernourished.

### Strengths and limitations

Our study emphasizes higher prevalence of vitamin D deficiency in critically ill children from a tropical country and its association with longer duration of ICU stay. Well defined eligibility criteria and prospective data collection are the strengths of our study. One major limitation is that we did not include a control group of healthy children. But data on this population is already available from the Indian sub-continent. The other important limitation is we only had the weight for age Z scores, which is not the best method to assess underlying nutritional status in children. Low levels of vitamin D are associated with inflammatory diseases. Whether they are the cause or the effect of vitamin D deficiency is unclear form the current evidence [32, 33]. Therefore, there is a need to cautiously interpret the results in patients with inflammatory conditions.

## Conclusions

We observed higher prevalence of vitamin D deficiency in critically ill children in our study population and the deficiency was associated with increased length of stay in the ICU. Our study results could be taken into account for designing interventional studies to study the outcomes of supplementation.

## Additional file

10.1186/s13613-015-0102-8: **Table S1.** Study definitions.
