# Supplementary material for: Vitamin D deficiency and length of pediatric intensive care unit stay: a prospective observational study
Source: Ann Intensive Care. 2016 Jan 8;6:3. doi: 10.1186/s13613-015-0102-8 (PMC4706541; doi:10.1186/s13613-015-0102-8)
Supplement: Supplementary file 1 — 10.1186/s13613-015-0102-8: Table S1. Study definitions. [file 13613_2015_102_MOESM1_ESM.docx]

**Supplemental Table 1. Study definitions**

| 1. Vitamin D deficiency was defined as serum 25 (OH) D levels of <20 ng/mL or <50 nmol/L[1]. 2. Hypocalcaemia was defined as an ionized value, 1.1 mmol/L [2]. 3. Need for mechanical ventilation was requirement of mechanical ventilation in the presence of abnormal ABG and/or evidence of neuromuscular weakness involving the respiratory muscles or hemodynamic instability. 4. Acute kidney injury was taken as an abrupt (within 48-hr) reduction in kidney function defined as an absolute increase in serum creatinine of more than or equal to 0.3 mg/dl, an increase in serum creatinine of more than or equal to 1.5 fold from baseline, or reduction in urine output (oliguria of less than 0.5 ml/kg per hour for >6-hr) [3]. 5. Severe undernutrition was defined as weight for age <-3SD as per WHO classification and moderate undernutrition as weight for age between -2 to -3SD [4]. 6. Pediatric Logistic Organ Dysfunction (PELOD) score included a total of 12 variables for six key organ dysfunctions (cardiovascular, respiratory, hematologic, neurologic, renal, and hepatic) [5]. 7. Pediatric Index of mortality- 2 score included eleven variables including pupillary reaction to light, systolic blood pressure, need for mechanical ventilation, base excess, PaO2/FiO2 ratio, elective admission and specified diagnosis calculated within 1 hour of admission [6]. 8. Total duration of ICU stay from the time of admission was calculated in days. |
| --- |

**References**

1. Holick MF, Binkley NC, Bischoff-Ferrari HA, et al.: Endocrine Society. Evaluation, treatment, and prevention of vitamin D deficiency: an Endocrine Society clinical practice guideline. *J Clin Endocrinol Metab*. 2011 ;96:1911-30. doi: 10.1210/jc.2011-0385.
2. Gauthier B, Trachtman H, Di Carmine F, et al. Hypocalcemia and hypercalcitoninemia in critically ill children. *Crit Care Med* 1990; 18:1215–1219.
3. Mehta RL, Kellum JA, Shah SV, Molitoris BA, Ronco C, Warnock DG, Levin A and the Acute Kidney Injury Network. Acute Kidney Injury Network: report of an initiative to improve outcomes in acute kidney injury. *Crit Care* 2007; 11: R 31.
4. WHO Multicentre Growth Reference Study Group. WHO Child Growth Standards based on length/height, weight and age. Acta Paediatr Suppl. 2006;450: 76–85.
5. Leteurtre S, Martinot A, Duhamel A, et al: Validation of the paediatric logistic organ dysfunction (PELOD) score: Prospective, observational, multicentre study. Lancet 2003; 362:192–197
6. Slater A, Shann F, Pearson G; Paediatric Index of Mortality (PIM) Study Group. PIM2: a revised version of the Paediatric Index of Mortality. Intensive Care Med. 2003; 29: 278-85.
